# Interactive video search tools: a detailed analysis of the video browser showdown 2015

**DOI:** 10.1007/s11042-016-3661-2

**Published:** 2016-07-23

**Authors:** Claudiu Cobârzan, Klaus Schoeffmann, Werner Bailer, Wolfgang Hürst, Adam Blažek, Jakub Lokoč, Stefanos Vrochidis, Kai Uwe Barthel, Luca Rossetto

**Affiliations:** 1 Klagenfurt University, Universitätstraße 65-67, 9020 Klagenfurt, Austria; 2 DIGITAL - Institute of Information and Communication Technologies, Joanneum research Forschungsgesellschaft mbH, Steyrergasse 17, A-8010 Graz, Austria; 3 Information and Computing Sciences, Utrecht University, Princetonplein 5, 3584 CC Utrecht, Netherlands; 4 SIRET research group, Department of Software Engineering, Faculty of Mathematics and Physics, Charles University in Prague, Malostranské nám. 25, 118 00 Prague, Czech Republic; 5 Centre for Research and Technology Hellas, Information Technologies Institute, 6th Klm Charilaou-Thermi Road, 57001 Thessaloniki, Greece; 6 Internationaler Studiengang Medieninformatik, Hochschule für Technik und Wirtschaft, Wilhelminenhofstr. 75a, D-12459 Berlin, Germany; 7 Department of Mathematics and Computer Science, University of Basel, Spiegelgasse 1, CH-4051 Basel, Switzerland

**Keywords:** Exploratory search, Video browsing, Video retrieval

## Abstract

Interactive video retrieval tools developed over the past few years are emerging as powerful alternatives to automatic retrieval approaches by giving the user more control as well as more responsibilities. Current research tries to identify the best combinations of image, audio and text features that combined with innovative UI design maximize the tools performance. We present the last installment of the Video Browser Showdown 2015 which was held in conjunction with the International Conference on MultiMedia Modeling 2015 (MMM 2015) and has the stated aim of pushing for a better integration of the user into the search process. The setup of the competition including the used dataset and the presented tasks as well as the participating tools will be introduced . The performance of those tools will be thoroughly presented and analyzed. Interesting highlights will be marked and some predictions regarding the research focus within the field for the near future will be made.

## Introduction

The *Video Browser Showdown (VBS)*, also known as Video Search Showcase, is an interactive video search competition where participating teams try to answer ad-hoc queries in a shared video data set as fast as possible. Typical efforts in video retrieval focus mainly on indexing and machine-based search performance, for example, by measuring precision and recall with a test data set. With video getting omnipresent in regular consumers lives, it becomes increasingly important though to also include the user into the search process. The VBS is an annual workshop at the International Conference on MultiMedia Modeling (MMM) with that goal in mind.

Researchers in the multimedia community agree that content-based image and video retrieval approaches should have a stronger focus on the user behind the retrieval application [13, 45, 50]. Instead of pursuing rather small improvements in the field of content-based indexing and retrieval, video search tools should aim at better integration of the human into the search process, focusing on interactive video retrieval [8, 9, 18, 19] rather than automatic querying.

Therefore, the main goal of the Video Browser Showdown is to push research on interactive video search tools. Interactive video search follows the idea of strong user integration with sophisticated content interaction [47] and aims at providing a powerful alternative to the common video retrieval approach [46]. It is known as the interactive process of video content exploration with browsing means, such as content navigation [21], summarization [1], on-demand querying [48], and interactive inspection of querying results or filtered content [17]. Contrarily to typical video retrieval, such interactive video browsing tools give more control to the user and provide flexible search features, instead of focusing on the *query-and-browse-results* approach. Hence, even if the performance of content analysis is not optimal, there is a chance that the user could compensate shortcomings through ingenious use of available features. This is important since it has been shown that user can give good performances even with very simple tools, e.g. a simple HTML5 video player [10, 12, 42, 44].

Other interesting approaches include using additional capturing devices such as the Kinect sensor in conjunction with human action video search [32], exercise learning in the field of healthcare [20] or interactive systems for video search [7]. In [7] for example, an interactive system for human action video search based on the dynamic shape volumes is developed – the user can create video queries by posing any number of actions in front of a Kinect sensor. Of course, there are many other relevant and related tools in the fields of interactive video search, video interaction, and multimedia search, which are however out of the scope of this paper. The interested reader is referred to other surveys in this field, such as [34, 46, 47].

In this paper we provide an overview of the participating tools along with a detailed analysis of the results. Our observations highlight different aspects of the performance and provide insight into better interface development for interactive video search. Details of the data set and the participating tools are presented, as well as their achieved performance in terms of score and search time. Further, we reflect on the achieved results so far, give detailed insights on the reasons why specific tools and methods worked better or worse, and subsume the experience and observations from the perspective of the organisers. Based on this, we make several proposals for highly promising approaches to be used with future iterations of this interactive video retrieval competition.

The remainder of the paper is organized as follows. Section 2 gives a short description of the competition. Section 3 makes an overview of both the presented tasks and of the obtained results. Section 4 provides short descriptions of the participating tools. A detailed analysis of the results for visual expert rounds is presented in Section 5. The results for the textual expert round are presented in Section 6 and the ones for the novice round in Section 7. A short historical overview over the last rounds of the Video Browser Showdown together with some advice on developing interactive video search tools are given in Section 8. Section 9 concludes the paper and highlights the most important observations stemming from the competition.

## Video browser showdown 2015

VBS 2015 was the fourth iteration of the Video Browser Showdown and took place in Sydney, Australia, in January 2015, held together with the International Conference on MultiMedia Modeling 2015 (MMM 2015). Nine teams participated in the competition and performed ten visual known-item search tasks (*Expert Run 1*), six textual known-item search tasks (*Expert Run 2*), as well as four visual and two textual known-item search tasks with non-expert users (*Novice Run*). The shared data set consisted of 153 video files containing about 100 hours of video content in PAL resolution (720 ×576@25p) from various BBC programs, and was a subset of the MediaEval 2013 Search & Hyperlinking data set [15]. The size of the data set was about 32 GB; the videos were stored in webm file format and encoded with the VP8 video codec, and the Ogg Vorbis audio codec. The data were made available to the participants about two months before the event.

During the event, users interactively try to solve search tasks with the participating tools; first in a closed expert session with the developers of a respective tool, then in a public novice session with volunteers from the audience – typically experts in the field of multimedia. Search tasks are so-called known-item search tasks where users search for information that they are familiar with. For the last two years [41] visual and textual queries were used. These are clips or textual descriptions of 20-seconds long target segments in a moderately large shared data set (100 hours at VBS 2015), which are randomly selected on site. After a clip or the text for a task is presented, participants have to find it in the database. They are presented through a PC connected to a projector, which runs the *VBS Server* that presents target segments (1) through playback of the corresponding clip for visual tasks, and (2) through presentation of a static textual description of the clip – collaboratively created by the organizers – for textual queries. After presentation of the visual or textual description, the VBS Server is responsible for collecting and checking the results found by the participants and for calculating the achieved *score* for each team.

The tools of all teams are connected to the VBS Server, and send information about found segments (frame numbers or frame ranges) to the server via HTTP requests. The server checks if the segment was found in the correct video and at the correct position and computes a score for the team, according to a formula that considers the search time (typically a value between 4 and 8 minutes) and the number of previously submitted wrong results for the search task (see [2, 42]). According to these parameters a team can get up to 100 points for a correct solved task, and in worst case zero points for a wrong or unanswered task. The scores are summed up and the total score of each session is used to determine the winner of the session. Finally, the team with the maximum grand total score is selected as the final winner of the competition. VBS 2015 used three sessions: (1) a visual expert’s session, (2) a textual expert’s session, and (3) a visual novice’s session.

In order to focus on the interactive aspects of search and avoid focusing too much on the automatic retrieval aspects, restrictions are imposed. Retrieval tools that only use text queries without any other interaction feature are therefore not allowed. However, participants may perform textual filtering of visual concepts, or navigate through a tree of textual classifications/concepts, for example. Moreover, the Video Browser Showdown wants to foster simple tools and, therefore, perform a novice session where volunteers from the audience use the tools of the experts/developers to solve the search tasks and by doing so test the usability in an implicit way.

In 2015, the focus of the competition has further moved towards dealing with realistically sized content collections. Thus, the tasks using only single videos, that were present in the 2013 and 2014 editions, have been discontinued, and the data set has been scaled up, from about 40 hours in 2014 to about 100 hours. The competition started with expert tasks in which visual and textual queries had to be solved. Then the audience was invited to join in and the tools were presented to allow the participants to understand how the tools are used by the experts. In the next sessions members of the audience (“novices”) took over for visual and text queries, and operated the tools themselves.

Each task in each of the three sessions (visual/textual expert run, novice run) aimed at finding a 20 seconds query video, where the excerpt does not necessarily start and stop at shot or scene boundaries. For visual queries, the video clip is played once (with sound) on a large, shared screen in the room. For textual queries, experts created descriptions of the contents of the clips, which were displayed on the shared screen and read to the participants. Participants were given a maximum time limit of eight minutes to find the target sequence in the corresponding video data (note that in the 2013 and 2014 competitions, the search in single videos was limited to three minutes, while the archive tasks in the 2014 competition had a limit of six minutes).

**Fig. 1:**
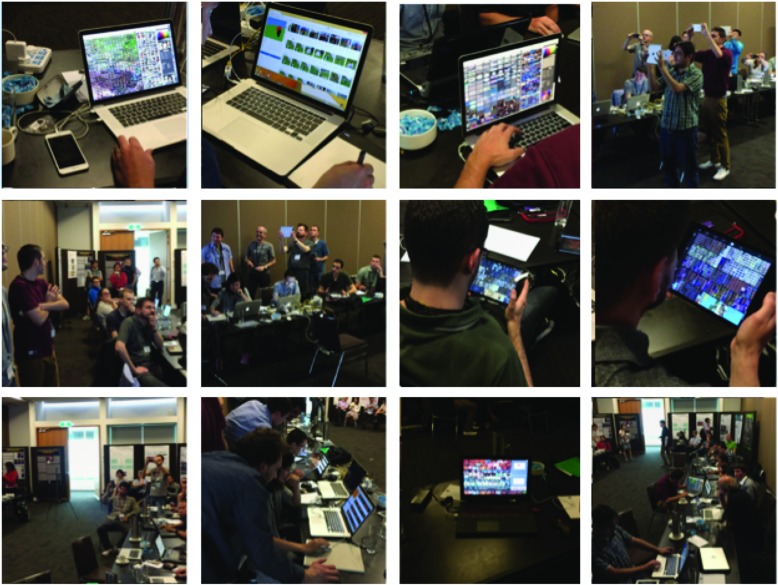
Teams competing during the VBS 2015 competition

The systems of all participating teams were organized to face the moderator and the shared screen, which was used for presenting the query videos and the current scores of all teams via the VBS server. Figure 1 shows the setup of the VBS session at MMM2015. The participating systems were connected to an HTTP-based communication server over a dedicated Wi-Fi private network. This server computed the performance scores for each tool and each task accordingly. Each tool provided a submission feature that could be used by the participant to send the current position in the video (i.e., the frame number or segment) to the server. The server checked the submitted frame number for correctness and computed a score for the corresponding tool and task based on the submission time and the number of false submissions. The following formulas were used to compute the score ${s_{i}^{k}}$ for tool *k* and task *i*, where ${m_{i}^{k}}$ is the number of submissions by tool *k* for task *i* and ${p_{i}^{k}}$ is the penalty due to wrong submissions:

1 $$ {s_{i}^{k}} = \frac{ 100 - 50\frac{t}{T_{max}} }{{p_{i}^{k}}},  $$

2 $$ {p_{i}^{k}}= \left\{\begin{array}{ll} 1, & \text{if } {m_{i}^{k}} \leq 1\\ {m_{i}^{k}} - 1, & \text{otherwise}. \end{array}\right.  $$

The overall score *S* ^*k*^ for tool *k* is simply the sum of the scores of all tasks of the three sessions. Equations (1) and (2) were designed in order avoid trial-and-error approaches: participants submitting several wrong results get significantly fewer points than participants submitting just one correct result. Additionally, the linear decrease of the score over time should motivate the teams to find the target sequence as fast as possible.

The hardware for the competition was not normalized; all participating teams were free to use the equipment best supporting the requirements and efficiency of their video browsers. The teams used notebook computers or tablets, depending on the respective browsing approaches.

## VBS2015 evaluation overview

The current section aims to give a general overview of the competition’s tasks and results and to point towards some of the most interesting conclusions. A detailed analysis and discussion of the results which focuses on the different tasks types follows in Section 5.

### Overview of the rounds and of presented tasks

As already mentioned in Section 2, the competition focused on two types of tasks, namely visual and textual tasks.

#### Expert run 1

In Table 1, an overview of the 10 target clips of the visual expert round (*Expert Run 1*) is presented as a series of temporally uniformly sampled frames captured at a two seconds interval. This should help readers to understand how the presented clips looked like. As visible in Table 1, some target clips showed quickly changing actions (e.g. tasks 1, 3, 6, 7, 8), only a few tasks - in particular 2 and 10 - showed scenes of longer duration, which are more distinct but proved hard to find.

**Table 1:** Overview of the presented video targets for the visual experts round

Task no.	Frame capture at									
	0s	2s	4s	6s	8s	10s	12s	14s	16s	18s
1										
2										
3										
4										
5										
6										
7										
8										
9										
10										

#### Expert run 2

The textual descriptions that the participants were provided with during the competition’s textual expert round (*Expert Run 2*) can be read in Table 2.

**Table 2:** Descriptions of the target segments provided for the text experts session

Task no.	Description
1	Panel of four participants with bluish background on the top (“COMEDIANS” displayed on their desk below) being asked a quiz question about a Russian exclave (i.e., separated region) in Europe, after the question is asked close-ups of the people are shown.
2	A man on a meadow (green grass in background), standing next to an ultralight aircraft and getting into a red and black overall.
3	A group of mostly kids practicing Karate moves indoors (in white clothes), including close-ups of a blond young woman talking to a girl, and shots showing the instructor, a bald man with glasses.
4	A prairie scenery with a hill on the left and mountains in the background, an old man with a black suit and hat walking slowly up the hill. He is first seen from behind, then a close-up of the man is shown. Then a close-up shot of a running wolf in the grass is shown.
5	A red/brown coloured map of Europe, with Alsace and the city of Strasbourg highlighted, showing also the surrounding countries (e.g., Germany, France). Then black/white shots of soldiers marching in a city (for several seconds). During the whole sequence a female sign language interpreter is visible in the lower right.
6	A BBC Four trailer, starting with a colourful huge bookshelf, then showing a sequence of countryside shots, and in each of them a yellow/gold glowing path showing music notes is appearing.

Table 3 shows an overview of the 6 target scenes described by Table 2 also as a sequence of temporally uniformly sampled frames at a two seconds interval. The difficulty with the textual tasks is the fact that the searchers have no idea about the actual visual presentation of the scene.

**Table 3:** Overview of the described video targets for the text experts round

Task no.	Frame capture at									
	0s	2s	4s	6s	8s	10s	12s	14s	16s	18s
1										
2										
3										
4										
5										
6										

#### Novice run

As already mentioned, the novice round that followed the visual and textual expert rounds, consisted of a total of six tasks, out of which four were visual tasks and two were textual tasks. They were presented as two sequences of two visual tasks followed by a textual task. Those tasks were extracted from the same pools as the tasks of the previous visual and text rounds and were in no way different.

The overview of the visual tasks (Task 1, Task 2, Task 4, Task 5) and that of the textual tasks (Task 3, Task 6) is shown in Table 4, while Table 5 gives the descriptions of the two textual tasks (Task 3 and Task 6 respectively).

**Table 4:** Overview of the presented and described video targets for the novices round

Task no.	rame capture at									
	”0	”2	”4	”6	”8	”10	”12	”14	”16	”18
1										
2										
3										
4										
5										
6

**Table 5:** Descriptions of the target segments provided for the text novices tasks

Task	Description
3	First a close-up of a beehive with many bees, then close-up shots of ants cutting and carrying large green leaves.
6	Piece about the ESA Ulysses mission, showing an image of the sun and the probe left above it, while zooming out it is explained how it orbits around the sun.
	The next shot shows the sun centered in a greenish hue (“STEREO” image). The flyby of a rendered model of the probe is shown.

### Overview of results

**Fig. 2:**
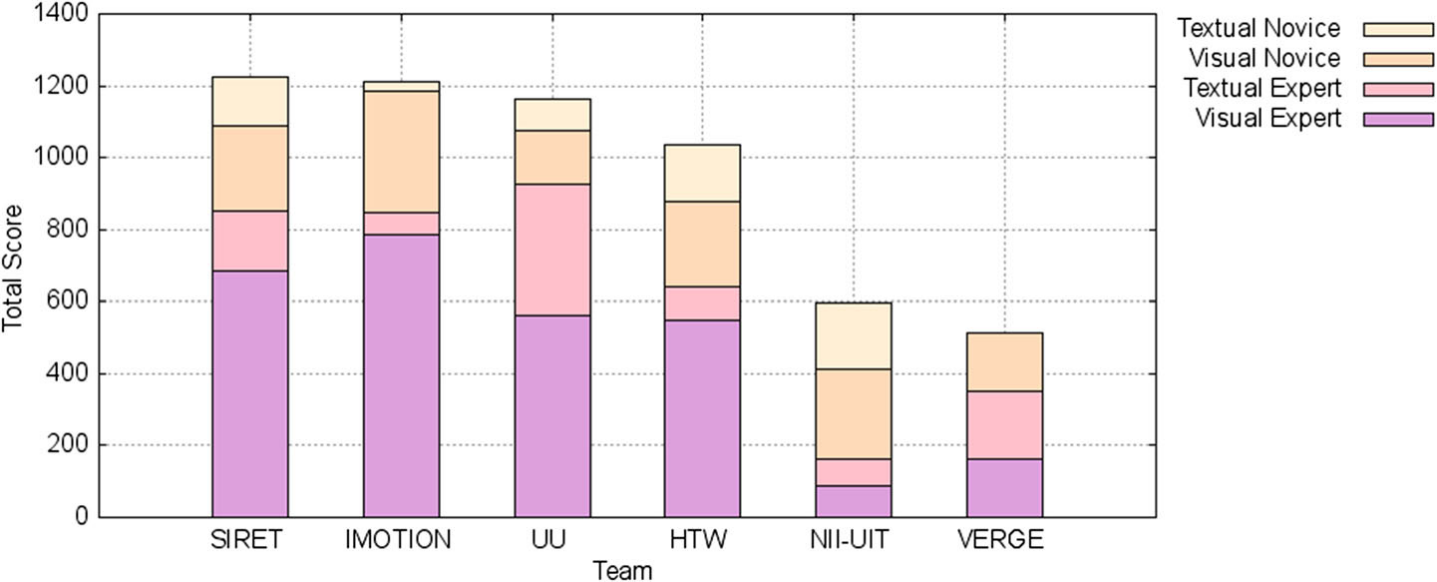
Total score of teams in the VBS 2015 competition

**Fig. 3:**
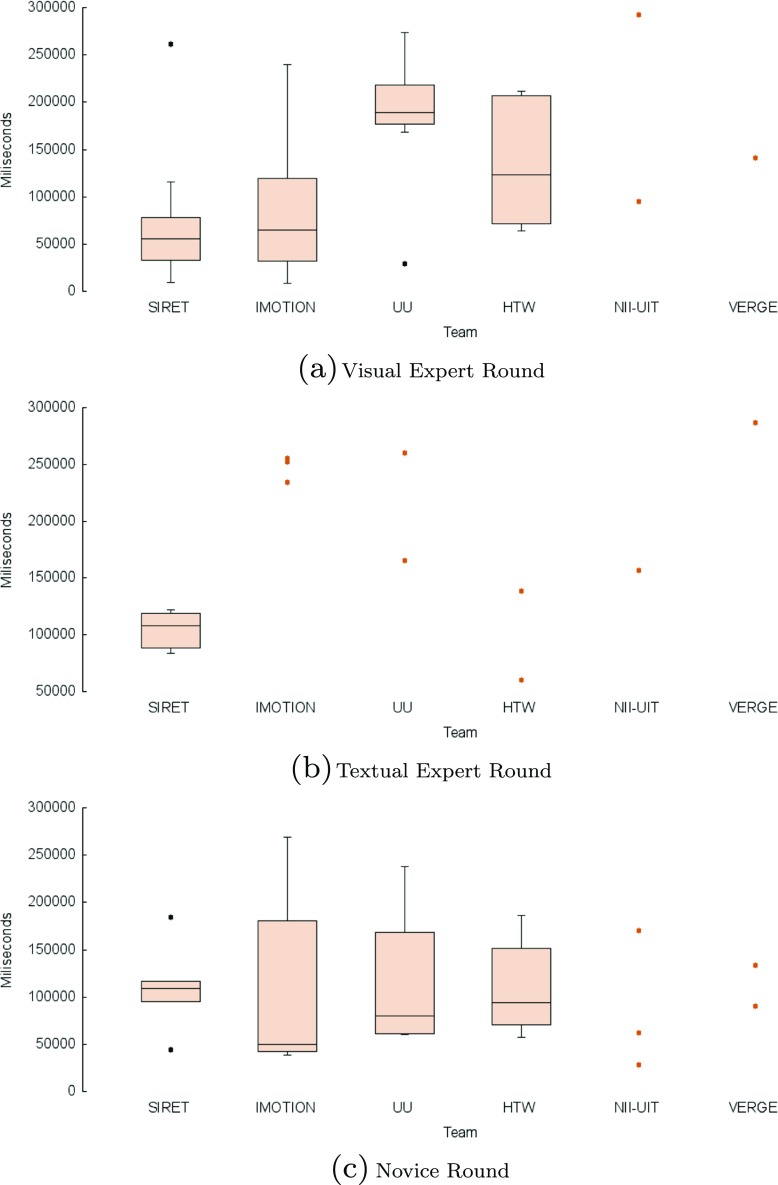
Box plot of the submission time per team in the VBS 2015 competition, based on correct submissions

**Fig. 4:**
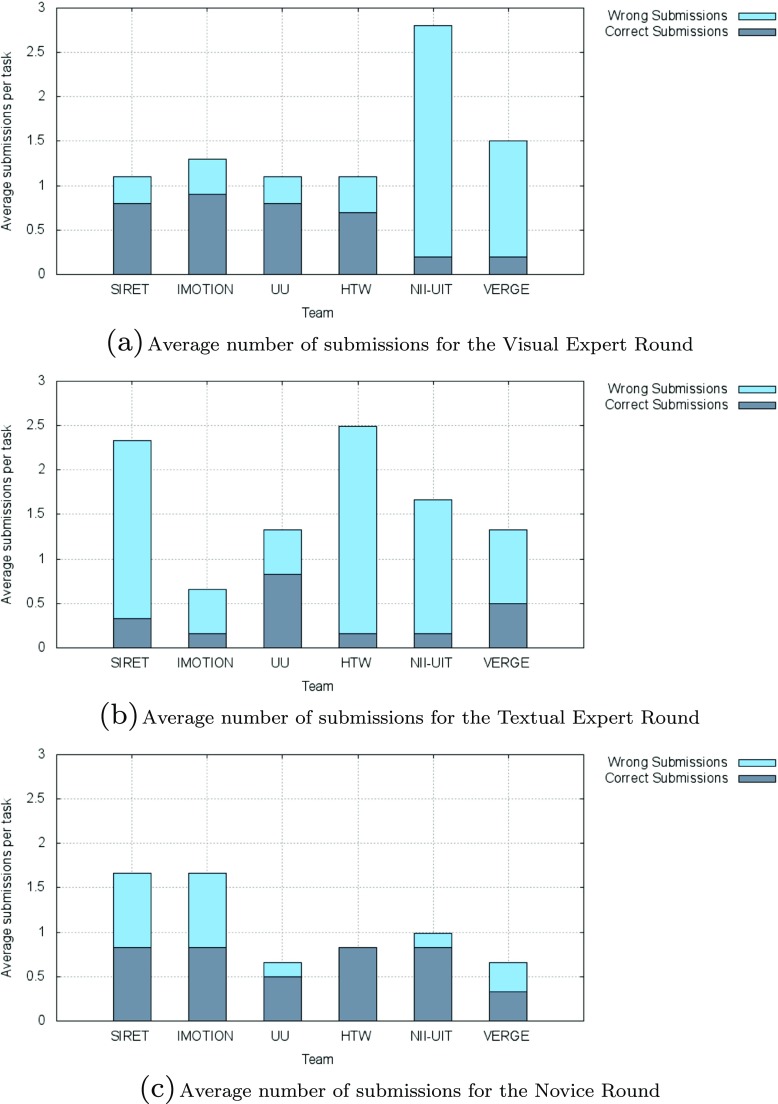
Average number of submissions (correct and wrong) in the VBS 2015 competition

In the following we present the results of the competition rounds. An overview of the final scores over all these rounds is presented in Fig. 2 while the overall submission times for the successful submissions within the competition across all tasks are shown in Fig. 3. The average number of submissions per round and team is shown in Fig. 4. The acronyms in all three figures’ legends identify the tools of the participating teams: HTW (Germany), IMOTION (Switzerland-Belgium-Turkey), NII-UIT (Vietnam - Japan), SIRET (Czech Republic), UU (The Netherlands), VERGE (Greece-UK). Detailed descriptions of those tools are available in Section 4, while the interested readers might consider the corresponding references in the Reference list for additional details.

Out of the nine participating teams [3, 5, 11, 22, 27, 35, 37, 39, 51], six managed to score points during the competition. Further analysis of the logs showed that one of the three non-scoring teams managed to solve one of the tasks but submitted its data using a wrong format.

Some interesting aspects can be observed when looking at both Figs. 2 and 3:

- The three top ranking teams (SIRET, IMOTION and UU) together with the forth (HTW) show the most uniform increases in terms of scored points across the visual expert and novice rounds (Fig. 2). In the case of the text expert round, only the UU and NII-UIT teams show this pattern. Overall the achievements during the novice round were over those in the text expert round.
- In the case of the NII-UIT team, the best scoring round was that of the novices (it actually won the round) during which the team climbed up to rank 5.
- The slowest of the participants (Fig. 2) was by far the UU team which was almost 2 times slower than the for-last participant in terms of speed - the HTW team. Since the UU team presented a tool designed for human computation this does not come as a surprise. What comes as a surprise is the excellent score they achieved - rank 3 overall. Also, it is interesting to note that the UU team was slowest during the visual expert round and got faster during the text expert and novice rounds with the additional note, that during the novice round only half of the targets were found (two visual and one textual targets).
- When comparing the three rounds, visual expert, textual expert and novice, the difference in speed, when it comes to finding the correct target, is not that big as when comparing experts and novices. The novices are a little bit slower except in the case of UU team, where the novices actually seem to perform faster then the experts. Unfortunately, due to the small number of novice tasks we are not able to generalize on whether this has to do with the actual tasks being presented, or this is because the novices just exploited the tools close to their full potential, as they had no false expectations. Also, as already mentioned, it is important to note that in most cases, the participants in the novice round were actually experts from the other participating teams which tested the “competition’s” tools.
- From the scoring point of view we see two team clusters: one that scored over 1000 points (SIRET, IMOTION, UU and HTW) and one that score under 600 (NII-UIT and VERGE), while from the time point of view, all the teams with the exception of the UU team, had similar overall completion times for their successful submissions.
  We have performed a one-way ANOVA to determine if the successful submission times for the visual experts round was different for the participating teams. Each of the IMOTION, SIRET and UU teams had one outlier. The search time was normally distributed for all interfaces, as assessed by Shapiro-Wilk’s test (p > .05). There was homogeneity of variances, as assessed by Levene’s test for equality of variances (p = .92). The search time was statistically significantly different between the interfaces, F(5, 30) = 3.045, p < .05.

## Scoring video search tools in VBS2015

### IMOTION

The IMOTION system [39] is a sketch and example-based video retrieval system. It is based on a content-based video retrieval engine called Cineast [38] that focuses on *Query-by-Sketch* and also supports *Query-by-Example* and *motion queries*.

In IMOTION, a user can specify a query by means of a sketch that may include edge information, color information, motion information, or any combination of these, or provide sample images or sample video snippets as query input. It uses multiple low-level features such as color- and edge histograms for retrieval.

The IMOTION system extends the set of features by high level visual features such as state-of-the-art convolutional neural network object detectors and motion descriptors. All feature vectors along with meta-data are stored in the database and information retrieval system ADAM [16] which is built upon PostgreSQL and is capable of performing efficient vector space retrieval together with Boolean retrieval.

The browser-based user interface, which is optimized to be usable with touch screen devices, pen tablets as well as a mouse, provides a sketching canvas as well as thumbnail previews of the retrieved results.

**Fig. 5:**
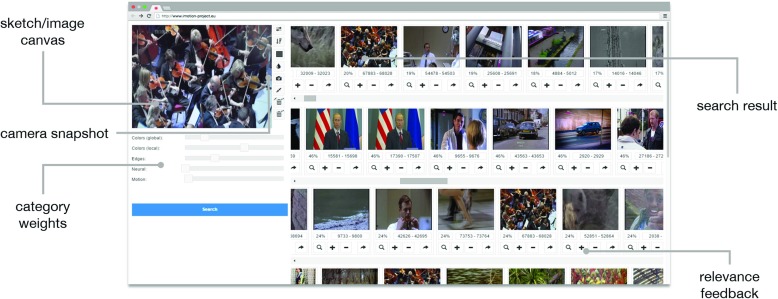
Screenshot of the IMOTION system

Figure 5 shows an example query with corresponding results. The results are grouped by row, each row containing shots which are similar to the query by a different measure such as colors, edges, motion or semantics. The topmost row shows the combination of these individual result lists whereas the influence of each category can be adjusted by sliders which change the combination in real time. The UI also offers a video capture functionality to collect reference frames using a webcam which then could be used during retrieval. Video capturing was successfully used during the visual tasks where images from the webcam were used as queries directly after cropping. In certain cases, the images were modified using the sketching functionality. For the textual challenges, only sketches were used.

### SIRET

**Fig. 6:**
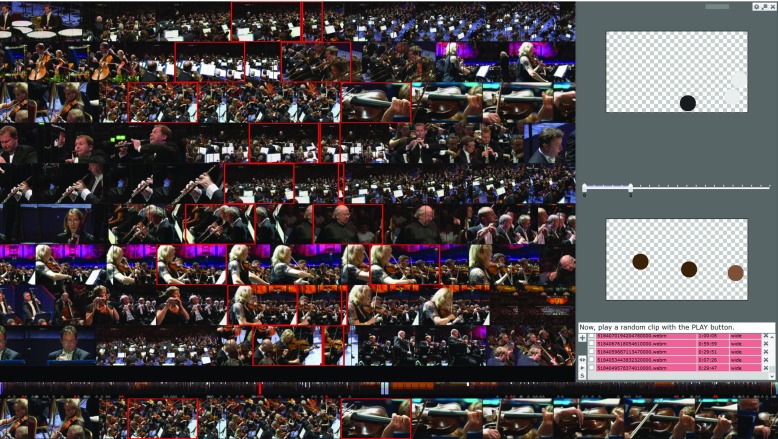
Screenshot of the SBVB tool in action

The SIRET system [5] is a new version of the Signature-based Video Browser tool (SBVB) [33] that was successfully introduced at the Video Browser Showdown in 2014. The tool combines two orthogonal approaches – efficient filtering using simple color-based sketches and enhanced presentation/browsing of the results. Both the filtering and the browsing parts of the application received various adjustments. Nonetheless, the overall concept utilizing position-color feature signatures [30, 40] was preserved, because representation of key-frames by the feature signatures enables effective and efficient location of searched key-frames. The concept relies on the *Query-by-Sketch* approach, where simple sketches representing memorized color stimuli can be quickly defined by positioning colored circles (see the right side of Fig. 6). The tool enables users to define either one sketch or two time-ordered sketches. In case when two sketches are specified, the tool searches for clips having matching key-frames in this particular order. The two searched key-frames have to be within a user specified time window. The retrieval model was described in more detail in [6]. The current enhanced version of the tool also considers the complexity of the key-frames to automatically adjust settings of the retrieval model.

Every query sketch adjustment is projected to the results area (see the left side of Fig. 6) immediately thanks to the efficient retrieval model employed which is based on position-color feature signatures. Each row represents one matched scene delimited by the matched key-frames (marked with red margin) and accompanied by a few preceding and following key-frames from the video clip. Any displayed scene can be selected as either positive or negative example for additional filtering. Alternatively, particular colored circles might be picked from displayed key-frames to the sketches similarly to picking up a color with the eyedropper tool. Regarding the video-level exploration, users may exclude a particular video from the search or contrary, focus on a single video. Especially the later mentioned feature often led to success as its appropriate usage can significantly increase results relevancy. When exploring a single video, users may find useful the extended results row (see the bottom of Fig. 6) enriched with Interactive Navigation Summary [43] displaying (in this case, 5 dominant colors of each key-frame).

### HTW

The HTW system [3] is a map-based browsing system for visually searching video clips in large collections. Based on ImageMap [4], it allows the user to navigate through a hierarchical-pyramid structure in which related scenes are arranged close to each other. An extended version of ImageMap can be viewed online at www.picsbuffet.com. The interaction is similar to map services like Google Maps: a view port revealing only a small portion of the entire map at a specific level. Zooming in (or out) shows more (or less) similar scenes from lower (or higher) levels. Dragging the view shows related images from the same level. While the hierarchical-pyramid of all scenes in the data set (“Map of Scenes”) has been precomputed to avoid performance issues, the map for a single video is generated on the fly and can therefore be filtered or altered based on the actions of the user.

The HTW-Berlin video browsing interface is divided into three parts: the browsing area on the left, the search result area in the middle and the search input area on the right.

**Fig. 7:**
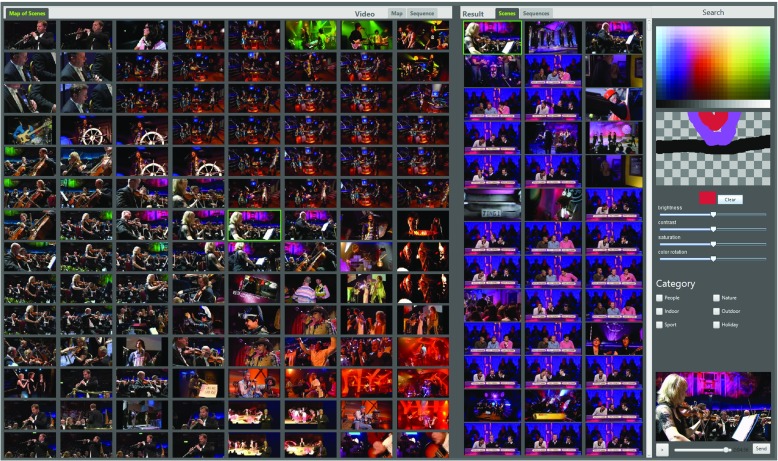
Screenshot of the HTW-Berlin tool

Generally the user starts with a sketch and maybe some adjustments to the brightness/contrast and saturation of the input. In the meanwhile the tool updates all views in real-time and presents the best match as a paused video frame on the bottom right of the interface (Fig. 7). The “Map of Scenes” jumps to a position where the frame of the video is located and other similar looking scenes are displayed in the result tab. If the detected scene is not the right one, the user can use the ImageMap to find related scenes and start a new search query by clicking them. All views are updated again and the sketch gets replaced by the selected frame. Upon finding the right scene it is suggested to check the “Video Map” for multiple look-alike alternatives and use the “Video Sequence” to verify the correct adjacent key frames.

In case the content of a scene is described as text or verbally, less or no visual information may be available, making a search nearly unfeasible. Usually the user still has an idea how the scene might look like. With the help of ImageMap it is possible to quickly navigate and check potential key frames.

### UU

The UU system [27] excludes all kinds of video analysis and machine-based query processing and relies exclusively on interaction design and human browsing abilities. Past research has demonstrated that a good and efficient interaction design can have a significant impact on search performance [24, 49] – especially when searching in single video files or small data sets. This claim is supported by previous years’ VBS results, for example, the baseline study presented in [42].

**Fig. 8:**
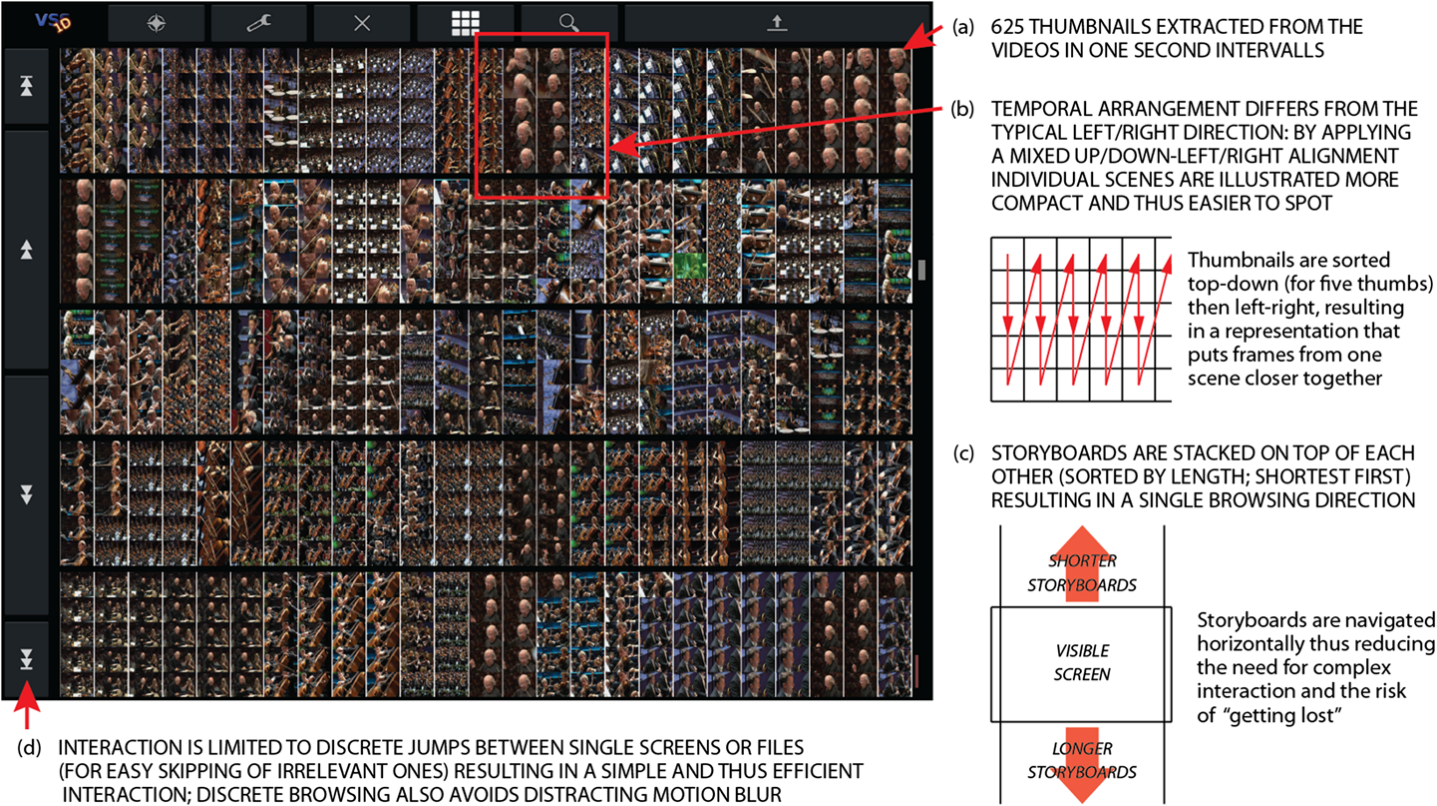
UU’s interface for purely human-based video browsing

Assuming that a simplistic design will increase search performance, all data is presented in a storyboard layout, i.e., a temporarily sorted arrangement of thumbnail images representing frames extracted from the videos. Considering that no video analysis is applied, these thumbnails have to be extracted at a low step size. Here, one second is used, resulting in about 360,000 single thumbnails for the approximately 100 hours of video. It is obvious that browsing such a huge amount of images in a short time is only possible if the related system is optimized for speed and the search task at hand. Figure 8 illustrates the related design decisions. Targeting a tablet as device with 9 inch screen size, and based on previous research about optimal images sizes for storyboards on mobiles [25, 26], 625 images are represented on one screen (cf. Fig. 8a). In order to better identify scenes, thumbnails are arranged not in the common left/right-then-top/down order but a mixture of up/down-left/right directions (cf. Fig. 8b). With 625 thumbnails on one screen and a total amount of about 360,000 thumbnails, more than 550 screens have to be visually inspected if the whole database has to be browsed. In order to speed up this process and considering related research results [23], interaction is simplified and restricted to up/down motions (i.e., storyboards of all files are represented horizontally; cf. Fig. 8c) and navigation is limited to discrete jumps between single screens or video files (cf. Fig. 8d).

### VERGE

The VERGE system [35] is an interactive retrieval system that combines advanced retrieval functionalities with a user-friendly interface, and supports the submission of queries and the accumulation of relevant retrieval results. The following indexing and retrieval modules are integrated in the developed search application: a) Visual Similarity Search Module based on K-Nearest Neighbour search operating on an index of lower-dimensional PCA-projected VLAD vectors [28]; b) High Level Concept Detection for predefined concepts by training Support vector machines with annotated data and five local descriptors (e.g. SIFT, RGB-SIFT, SURF, ORB etc), which are compacted and aggregated using PCA and encoding; the output of the trained models is combined by means of late fusion (averaging); c) Hierarchical Clustering incorporating a generalized agglomerative hierarchical clustering process [29], which provides a structured hierarchical view of the video keyframes.

**Fig. 9:**
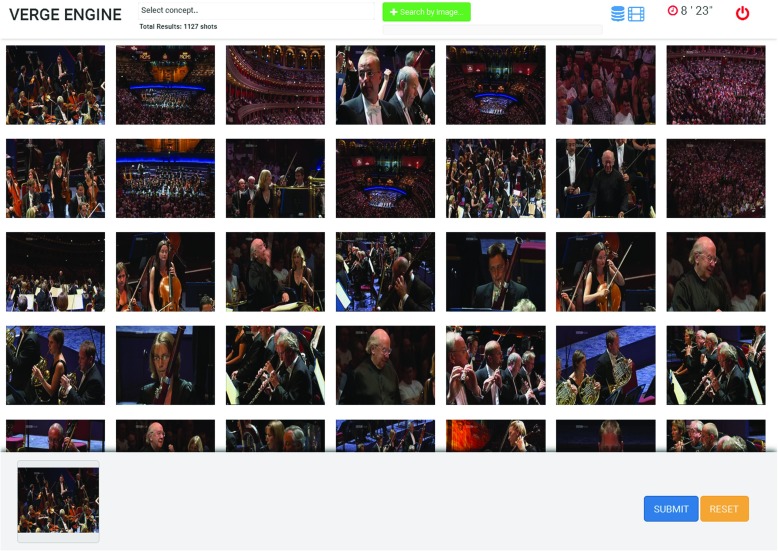
VERGE video retrieval engine interface

The aforementioned modules allow the user to search through a collection of images and/or video keyframes. However, in the case of a video collection, it is essential that the videos are preprocessed in order to be indexed in smaller segments and semantic information should be extracted. The modules that are applied for segmenting videos are: a) Shot Segmentation; and b) Scene Segmentation. All the modules are incorporated into a friendly user interface (Fig. 9) in order to aid the user to interact with the system, discover and retrieve the desired video clip.

## Evaluation details on visual experts tasks

### Search time and achieved points

**Fig. 10:**
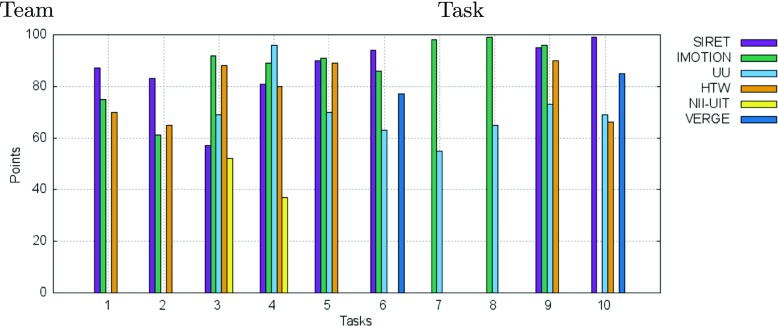
Breakdown per individual tasks of the scores for the visual experts round

**Fig. 11:**
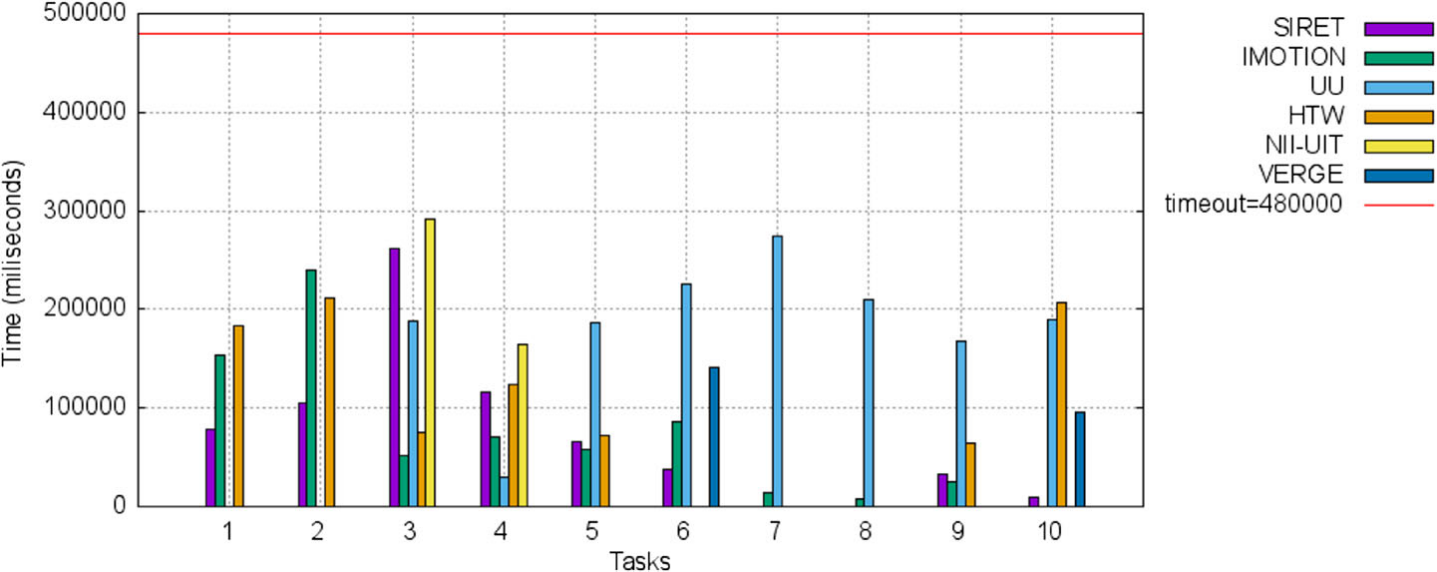
Breakdown per individual tasks of the time needed for the successful submissions for the visual experts round

The breakdown of the final scores per task as well as the information regarding the time needed for the completion of each successful submission are presented in Figs. 10 and 11.

The SIRET team completed 8 out of 10 proposed tasks, while the IMOTION team successfully completed 9 of the 10 tasks. The UU and HTW teams completed 7 tasks while the NII-UIT and VERGE teams completed 2 tasks each.

From Table 6 we can see that these two teams (in particular NII-UIT) submitted many wrong results, which however were quite visually similar to the targets. The frames for all the false submissions are shown in Table 6 as thumbnails with red contour. It can be seen that, most of the times, the visual similarity when compared with the target scenes is very high (see both Tables 1 and 7 - the thumbnails with the green contour). This is because the majority of the tools concentrated on the visual features, which in cases of similar/identical looking frames from different segments/shots does not suffice for correctly identifying the target scene. In those cases, additional information like for example the audio track, is needed.

**Table 6:** Frames submitted during the visual experts round (wrong submissions have red contours; right submissions have green contours)

Team	Task									
	1	2	3	4	5	6	7	8	9	10
SIRET										
SIRET										
										
										
IMOTION										
										
UU										
										
HTW										
										
										
NII-UIT										
										
										
										
										
										
										
VERGE										
										
										
										
		...

**Table 7:** Frames submitted by the participant teams during the textual experts round (wrong submissions have red contours; right submissions have green contours)

Team	Task							
	1			2	3	4	5	6
SIRET								
								
								
IMOTION								
UU								
								
								
HTW								
								
								
								
								
NII-UIT								
								
								
								
VERGE								
								

A closer examination of Fig. 11 hints towards some interesting aspects:

- The IMOTION team had a slow start during the first 2 tasks, but then submitted the correct target scene very quick for the next 7 tasks. In fact they were the quickest for task number 3, 5, 7, 8 and 9.
- The same slow start during the first tasks can be seen in the case of the other teams: SIRET, HTW and UU. This might be due to an accommodation phase in which the teams got accustomed to the competition spirit as well as with the responsiveness of the various tools’ features under the on-site conditions.
- In the case of the three teams that successfully completed the first two tasks IMOTION, SIRET and HTW, the time needed to complete the tasks actually increases: this can be explained either by the fact that the target scene is located “deep” within the archive and more time is needed for investigation, or by the fact that they tried to apply for the second round the same strategy they employed for the first one and failed.
- For the UU team which had a tool that relied heavily on human computation, the time needed to successfully find a target scene shows the lowest variance with the exception of task 4 in which actually the UU team was the fastest (this might be due to the positioning of the target scene at the very beginning of the video and the navigation model employed).
- The tasks 3, 4 and 6 were successfully completed by 5 teams; in the case of tasks 3 and 4 by the same teams configuration: IMOTION, SIRET, UU, HTW and NII-UIT. The tasks 5, 9 and 10 were completed by 4 teams, tasks 1 and 2 by 3 teams while tasks 7 and 8 were completed by only 2 teams: IMOTION and UU. While in the case of IMOTION it seems that the internals have played the most important role, since the team was fastest for exactly those two “difficult” tasks, in the case of UU it seems to be raw human power that had been rewarded - in the case of task 7 the UU team had the slowest completion time over all tasks (not in comparison with the other teams though).

### Erroneous Submissions

It is interesting to note that the IMOTION and UU teams always identified the correct files, while the HTW and SIRET teams each had 1, and 2 wrongly identified files respectively, but in all 3 cases the correct file was later identified. The UU team achieved the best ratio for correct submissions vs. wrong submissions with 8 correct submissions to 3 wrong submissions.

**Fig. 12:**
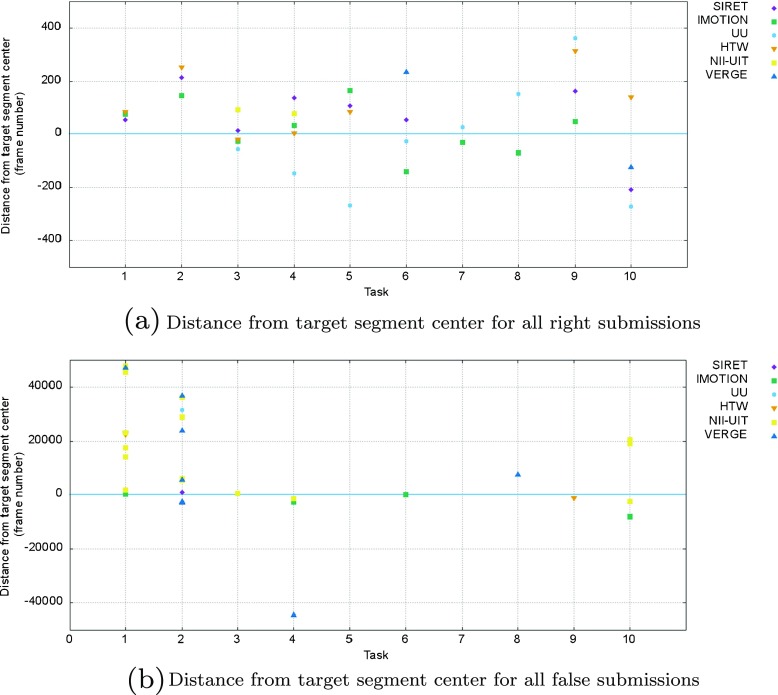
Breakdown per tasks for teams’ submissions distances from target segment center for visual experts session

The distances in terms of frame numbers from the submitted segment center to the target segment center for both right and false submissions are presented in Fig. 12 (Fig. 12a for right/successful submissions and Fig. 12b for false submissions within the correct file). Negative values in both sub-figures, represent submissions in the first half of the target segment or frames leading up to the target segment, while positive values represent submissions in the second half of the target segment or frames past the target segment up to the end of the video.

In the case of the successful submissions (see Fig. 12a) a greater number of submissions have been issued with frames from the second half of the target segment (positive values), although there is also a significant number of submissions from the first half. In the case of the false submissions (Fig. 12b) most of them are made by indicating frames/segments past the target segment and later in the video.

The actual frames sent for validation by the participating teams, for all the successful submissions, can be seen in Table 6 as thumbnails with green contour (in the case of the IMOTION team which sent a frame range, as permitted by the competition rules, we have chosen the central frame of the sent segment).

## Evaluation details on textual experts tasks

The final scores at the end of the text round are also shown in Fig. 2. This proved to be the most challenging round of the competition. From the nine participating teams, only the UU team managed to score more than 50 % of the possible points for the session, with 367 points out of 600, while VERGE and SIRET scored close to 33 % with 188 and 166 points respectively. The performance of the UU team is particularly surprising since it employed only human computation and only static small thumbnails (no audio or video playback capabilities). Those results show there is still enough room for improvement in this area.

**Fig. 13:**
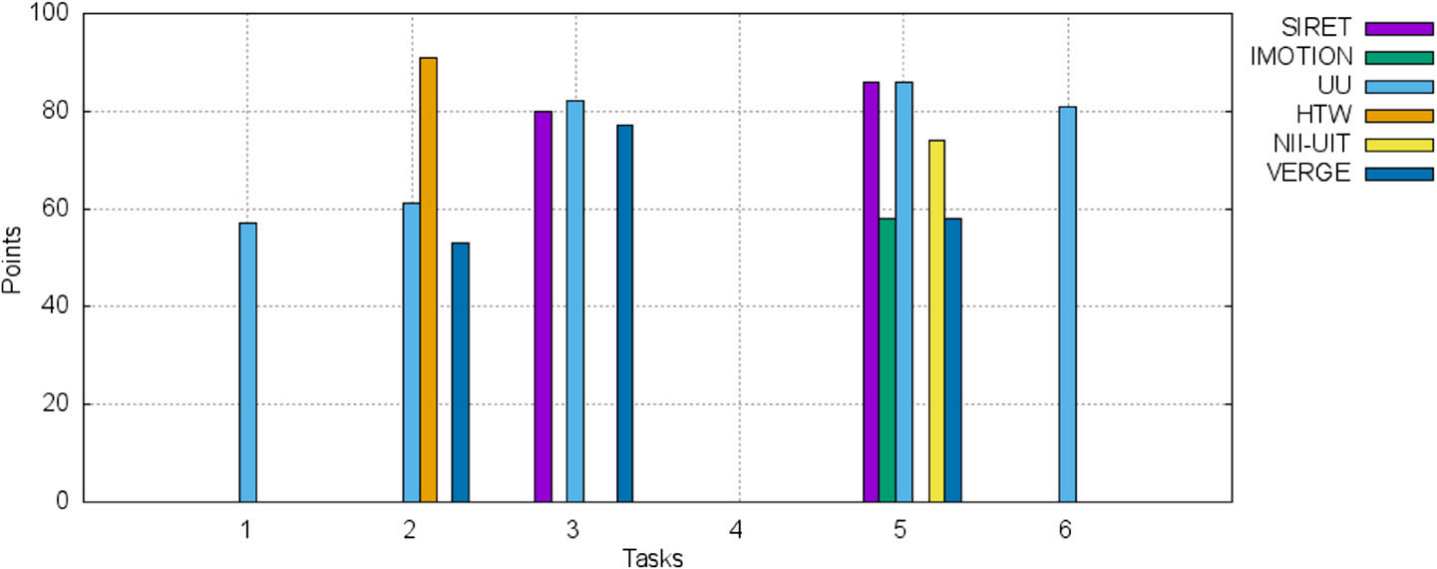
Breakdown per individual tasks of the scores for the text experts round

**Fig. 14:**
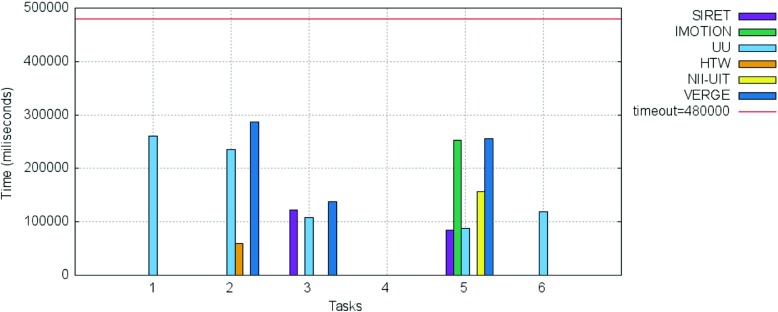
Breakdown per individual tasks of the time needed for the successful submissions for the text experts round

Figures 13 and 14 show the breakdown of the final scores per task as well as the information regarding the time needed for the completion of each successful submission for the tasks in the text experts’ round.

Regarding task completion, no team managed to solve Task 4, while Task 1 and Task 6 were solved only by the UU team. Task 2 and Task 3 were successfully solved by 3 teams, while Task 5 seams to have been the easiest, with 5 teams solving it. All teams scored over 50 % of the available points per task (more than 50 points) and as in the case of the visual round, no successful submission was made past the 5 minutes mark.

## Evaluation details on the novice tasks

Figure 2 also shows the scores obtained by each of the participating teams for the visual and text tasks of the novice round. With four visual tasks and two text tasks, the maximum possible scores were 400 for the visual and 200 for the text tasks. This gave an overall of 600 possible points for the novice round, as much as the text expert round.

IMOTION obtained the highest score for the visual novice (340 points, close to the maximum of 400), but the lowest score (28 points from the maximum of 200) for the text novice. NII-UIT, HTW, SIRET, VERGE and UU also scored high in the visual novice tasks. For the text novice tasks, NII-UIT, HTW and SIRET obtained the highest scores, with NII-UIT scoring a surprisingly high score of 181 points. The UU team also managed to score 90 points while, as already mentioned, IMOTION were last in this category with only 28 points. VERGE scored no points for the text tasks in the novice round, which is very surprising, since their concept-based search tool seems to be particularly well suited for novices.

**Fig. 15:**
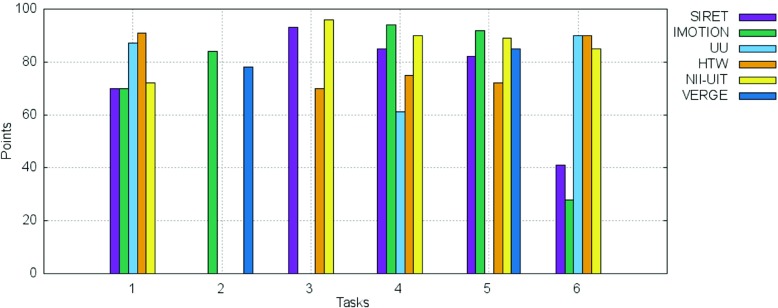
Breakdown per individual tasks of the scores for the novices round for both visual and textual tasks

**Fig. 16:**
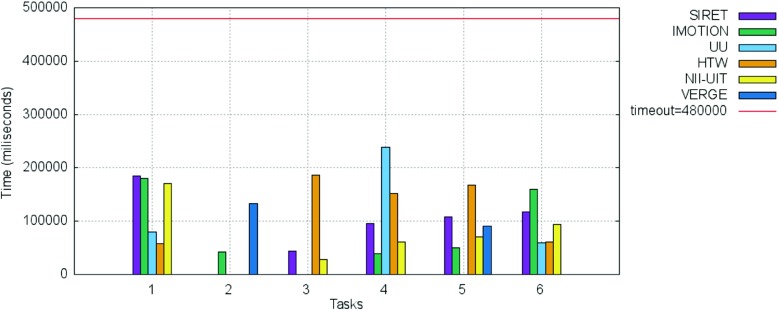
Breakdown per individual tasks of the time needed for the successful submissions for novice round for both visual and textual tasks

The breakdown per tasks of the scores for the novice round as well as the time needed for the correct submissions are shown in Figs. 15, and 16 respectively. The scores obtained for all 4 visual tasks (Task 1, Task 2, Task 4, Task 5) were high and very high for all the novices - all scored over 60 points. This was true also for the 2 text tasks (Task 3 and Task 6), with the 2 notable exceptions of the IMOTION and SIRET teams in the case of Task 6 for which both achieved under 50 points. When looking at the time needed for submitting a correct answer as shown in Fig. 16, it can be seen that it was way under half of the maximal available time in most of the cases.

Overall, as also seen from Fig. 4c which presents the average number of submissions (wrong as well as correct) per team for the novice round, the participants seemed more than cautious when submitting frames for validation. In fact the novice round had overall the smallest number of wrong submissions when compared with both expert rounds. We have two possible non-exclusive explanations for this:

- it was the final round which was to decide the winner in a very tight competition and the participants were over-cautious;
- the majority of the“novices” were in fact members of the participating teams testing the “competition’s” tools under their colleagues close supervision and they did not want to “sabotage” their winning chances by making wrong submissions and by this achieving a low score. At that point we want to mention that the novice session in general is kind of problematic for the final analysis, as it may distort the results. Therefore, we might want to skip it in future iterations of the VBS.

A closer inspection revealed that there is no difference between the two types of tasks (visual vs. text) from the outcome of submissions point of view. It can be seen though, that Task 6 seemed more difficult since it had overall the largest number of false submissions in both the right and wrong files. It has also been a textual task. The unusual large number of submissions for Task 6 when compared to the other five previous tasks could be also explained by the fact that it was the last task and by an all-or-nothing approach from some of the participants. In fact, the winner of the competition was decided by novices during this last task, when SIRET overcame IMOTION, after three false submissions for each team: two vs. one from a wrong file for SIRET when compared to IMOTION. The difference was made by the speed of the submissions, with SIRET being twice as fast as IMOTION for this particular task with 117049 ms vs. 268769 ms.

## Development of interactive video search tools

VBS sessions happen to be indeed interactive and VBS 2015 was not an exception. Participants are exploring tools of other teams and the audience often discuss the approaches during breaks etc. It is thus natural to adapt and perhaps enhance a well-performing feature introduced by some other participant. Thanks to the gradual improvements, a tool winning the competition one year would probably fail without further development the next year. Several teams participated steadily for the last few years, each year improving their tools, adding modalities, features or ever reworking their concepts from scratch. We may ask then, are there any trends that we can distinguish? Can we find a common feature that is sooner or later incorporated by almost every team? And lastly, can we derive guides or best practices for developing such interactive video search tool?

In the following paragraphs, we track the evolution of the tools which had won VBS in one of the previous years, namely teams AAU (2012), NII-UIT (2013) and SIRET (2014 and 2015).

NII-UIT established themselves in 2013 by actually winning VBS [31]. The tool utilized filtering by prior-detected concepts and visual content, a grid of dominant color more precisely. The results were presented with a coarse-to-fine hierarchical refinement approach. In 2014 they came up with quite a similar tool with one important enhancement – a user could define a sequence of patterns, i.e., define two sets of filters and search for clips having two matching scenes in the same order [36]. Finally, in 2015 they additionally focused on face and upper body concepts together with audio filters and replaced the grid of dominant colors with less rigid free-drawing canvas [37].

The tool which was introduced by SIRET team in 2014 [33] appeared quite different. Instead of complex processing pipelines, such as state-of-the-art concept detectors etc., the authors employed only one feature capturing color distribution of key-frames (so called *feature signatures*) together with convenient sketch drawings (apparently, NII-UIT adapted sketch drawings later on). Surprisingly it was enough to win the VBS that year. Note that similarly to NII-UIT’s tool, users were allowed to specify two consecutive sketches to improve the filtering power which seemed to be quite effective. Changes introduced to the tool in 2015 [5] were rather subtle, focusing the browsing part of the tool, such as compacting static scenes in order to save space etc.

The list of winners of previous VBS competitions is completed by AAU tool from 2012 [14] which is somewhat similar to the most recent UU’s application, both exhibiting surprisingly powerful human computation potential. In this case, the videos are simply scanned in parallel during the search time without any prior content analysis.

Overlooking all the various approaches, we can identify three main techniques appearing repetitively:

- Content-based filtering may be based on either high-level concepts or low-level features. In particular, most of the participants used some kind of color-based filtering and although their filtering power decreases steadily as the dataset size increases, they seem to remain quite effective. Also, temporal filtering (e.g., two content sketches that focus two neighboring segments in a video) seem to be quite effective, because static approaches (focusing on a single image only) do not work well with the sheer amount of frames.
- Browsing is perhaps a crucial part that cannot be avoided. In many cases, the number of relevant results is simply too large to fit one screen and users need an effective and convenient way to browse through the results.
- As users do so, they will probably encounter scenes quite similar to the searched one which may be used as a query for additional similarity search. By giving the search engine either positive or negative examples users can rapidly navigate themselves towards the target if an appropriate similarity model is employed. Note that regarding the textual tasks, we face the problem of proper initialization of this similarity search loop.

At this moment, we do not see a single approach, feature or concept that is clearly outperforming the others. We believe, though, that a successful interactive video search tool has to incorporate all the three techniques mentioned above.

It is of course hard to predict the future in this challenging field. However, we can assume that future systems will also strongly rely on color-based filtering (e.g., color maps such as used by the system described in Section 4.3), on concept-based filtering (e.g., visual semantic concepts detected with deep learning approaches), on temporal filtering as well as on improved content visualization with several techniques, for example with hierarchical refinement of similar results. Since the VBS plans to increase the size of the data set every year, we believe that in the long run the biggest challenge will be the efficient handling of the large amount of content, i.e., content descriptors and indexes, and providing a highly responsive interactive system that allows for iterative refinement.

## Conclusions

In the context of the discussion that follows, we would like to highlight the fact that all target segments for the three rounds, were randomly generated from the 154 files totaling over 100 hour of video material that formed the competition dataset. Approximately 10% of the cues had also textual description assigned by the organizers. From within those two pools, the target videos for the competition rounds were randomly chosen: ten targets for the visual expert round, six targets for the text expert round and six targets for the novice round (four targets for visual tasks and two targets for text tasks).

The case of the novice round differs a little bit from the two expert rounds, because the visual and textual tasks were mixed and not consecutive. Also, because of time constraints, the organizers were able to allow only six novice tasks out of which four were visual (Task 1, Task 2, Task 4 and Task 5) and two were textual tasks (Task 3 and Task 6).

Some interesting facts emerge when looking and comparing the figures presenting the breakdowns per individual tasks of the scores and of the times needed for the correct submissions for the three rounds:

- The visual tasks in the novice round achieved the best overall scores across all teams when compared with the visual tasks in the visual expert round.
- the text tasks in the novice round (2 tasks) achieved comparable results with the best performances across the 6 tasks in the text expert round.
- The completion time in the case of the visual and text tasks in the novice round is comparable with the completion time in the case of the visual and text expert rounds. The main difference is that the advantage that the SIRET, IMOTION and sometimes UU teams had in the expert rounds, is much reduced in the case of the novice round.
- The novice round brought the best performance for the NII-UIT team in both scored points and speed. Actually for the first text task in the novice round, the NII-UIT team achieved the best score and had the fastest correct submission.

It is also interesting to have a closer look at the frames being submitted across the three competition rounds, both the ones of the correct submissions as well as the ones of the wrong submissions (both from the correct and wrong files) and to compare them with the uniform sampled frames of the video targets. The tables in question are Table 1, Table 1 and Table 4 for the overview of the target videos and Tables 6 and 7 for the correct submitted frames as well as for the wrong submitted frames. Some interesting observations can be made:

- Within each of the scenes used as targets in the visual expert round there are multiple highly similar images (this is also apparent in Table 1 as well as Tables 3 and 4 which display overviews of the 20 seconds long target scenes while using 2 second granularity for each image). Because of the granularity used in the figures, not all the details are visible, from here the difference in terms of actually submitted frames.
- The scenes are very diverse including indoor and outdoor shots as well as overlays of computer generated content spread across TV reporting, TV series, TV documentaries.

The best results were obtained by tools that employed some form of sketching for an query-by-example approach, as in the case of the SIRET and IMOTION teams, or that made heavy use of browsing, like in the case of the UU team which had an approach centered on human computation. All those tools had effectively put the user in the center of their approaches to an interactive multimedia retrieval system and had tried to exploit its mental and physical capacities to their fullest in order to solve the proposed tasks. The results of the text tasks during both the expert and novice rounds show that there is still a lot of room for improvement and that in this particular case further research is needed.

## References

[1] Adams B, Greenhill S, Venkatesh S (2012) Towards a video browser for the digital native. In: ICMEW’12. doi:10.1109/ICMEW.2012.29, pp 127–132

[2] Bailer W, Schoeffmann K, Ahlström D, Weiss W, Del Fabro M, Mei T (2013) Interactive evaluation of video browsing tools. In: Li S, Saddik A, Wang M, Sebe N, Yan S, Hong R, Gurrin C (eds) Advances in multimedia modeling, lecture notes in computer science. doi:10.1007/978-3-642-35725-1_8, vol 7732. Springer, Berlin Heidelberg, pp 81–91

[3] Barthel KU, Hezel N, Mackowiak R (2015) Graph-based browsing for large video collections. In: He X, Luo S, Tao D, Xu C, Yang J, Hasan M (eds) MultiMedia modeling, lecture notes in computer science. doi:10.1007/978-3-319-14442-9_25, vol 8936. Springer International Publishing, pp 237–242

[4] Barthel KU, Hezel N, Mackowiak R (2015) Graph-based browsing for large video collections. In: Proceedings of multimedia modeling - 21st international conference, MMM 2015. Part II. doi:10.1007/978-3-319-14442-9_21. Sydney, pp 237–242

[5] Blažek A, Lokoč J, Matzner F, Skopal T (2015) Enhanced signature-based video browser. In: Proceedings of multimedia modeling - 21st international conference, MMM 2015. Part II. Sydney, pp 243–248

[6] Blažek A, Lokoč J, Skopal T (2014) Video retrieval with feature signature sketches. In: Proceedings of similarity search and applications - 7th international conference, SISAP 2014. Los Cabos, pp 25– 36

[7] Chen HM, Cheng WH, Hu MC, Lin YC, Hsieh YH (2013) Human action search based on dynamic shape volumes. In: Li S, Saddik AE, Wang M, Mei T, Sebe N, Yan S, Hong R, Gurrin C (eds) MultiMedia modeling, lecture notes in computer science, vol 7733. Springer International Publishing, pp 99–109

[8] Christel M, Huang C, Moraveji N, Papernick N (2004) Exploiting multiple modalities for interactive video retrieval. In: ICASSP’04. doi:10.1109/ICASSP.2004.1326724, vol 3, pp iii–1032

[9] Christel MG, Yan R (2007) Merging storyboard strategies and automatic retrieval for improving interactive video search. In: CIVR’07. doi:10.1145/1282280.1282351. ACM, New York, pp 486–493

[10] Cobârzan C (2014) Evaluating interactive search in videos with image and textual description defined target scenes. In: IEEE international conference on multimedia and expo workshops (ICMEW), 2014. IEEE, pp 1–6

[11] Cobârzan C, Del Fabro M, Schoeffmann K (2015) Collaborative browsing and search in video archives with mobile clients. In: He X, Luo S, Tao D, Xu C, Yang J, Hasan M (eds) MultiMedia modeling, lecture notes in computer science, vol 8936. Springer International Publishing, pp 266– 271

[12] Cobârzan C, Schoeffmann K (2014) How do users search with basic html5 video players? In: Gurrin C, Hopfgartner F, Hurst W, Johansen H, Lee H, O’Connor N (eds) MultiMedia modeling, lecture notes in computer science, vol 8326. Springer International Publishing, pp 109–120

[13] Datta R, Joshi D, Li J, Wang JZ (2008). Image retrieval: ideas, influences, and trends of the new age. ACM Comput Surv.

[14] Del Fabro M, Boszormenyi L (2012) Aau video browser: Non-sequential hierarchical video browsing without content analysis. In: Schoeffmann K, Merialdo B, Hauptmann AG, Ngo CW, Andreopoulos Y, Breiteneder C (eds) MultiMedia modeling, lecture notes in computer science, vol 7131. Springer International Publishing, pp 639–641

[15] Eskevich M, Aly R, Chen S, Jones GJF (2013) The search and hyperlinking task at mediaeval 2013. In: Proc. of MediaEval Workshop, pp 18–19

[16] Giangreco I, Al Kabary I, Schuldt H (2014) Adam-a database and information retrieval system for big multimedia collections. In: IEEE international congress on big data (bigdata congress), 2014. IEEE, pp 406–413

[17] Girgensohn A, Shipman F, Wilcox L (2011) Adaptive clustering and interactive visualizations to support the selection of video clips. In: ICMR ’11. doi:10.1145/1991996.1992030. ACM, New York, pp 34:1–34:8

[18] Hopfgartner F (2007) Understanding video retrieval. VDM Verlag

[19] Hopfgartner F, Urban J, Villa R, Jose JM (2007) Simulated testing of an adaptive multimedia information retrieval system. In: CBMI’07, pp 328–335

[20] Hu MC, Chen CW, Cheng WH, Chang CH, Lai JH, Wu JL (2015). Real-time human movement retrieval and assessment with kinect sensor. IEEE Trans Cybern.

[21] Huber J, Steimle J, Mühlhäuser M (2010) Toward more efficient user interfaces for mobile video browsing: an in-depth exploration of the design space. In: MM’10. doi:10.1145/1873951.1873999. New York, pp 341–350

[22] Hudelist MA, Xu Q (2015) The multi-stripe video browser for tablets. In: He X, Luo S, Tao D, Xu C, Yang J, Hasan M (eds) MultiMedia modeling, lecture notes in computer science, vol 8936. Springer International Publishing, pp 272–277

[23] Hürst W, Darzentas D (2012) Quantity versus quality: The role of layout and interaction complexity in thumbnail-based video retrieval interfaces. In: Proceedings of the 2Nd ACM international conference on multimedia retrieval, ICMR ’12. doi:10.1145/2324796.2324849. ACM, New York, pp 45:1–45:8

[24] Hürst W, Hoet M (2015) Sliders versus storyboards – investigating interaction design for mobile video browsing. In: He X, Luo S, Tao D, Xu C, Yang J, Hasan M (eds) MultiMedia modeling, lecture notes in computer science. doi:10.1007/978-3-319-14442-9_11, vol 8936. Springer International Publishing, pp 123–134

[25] Hürst W, Snoek C, Spoel WJ, Tomin M (2011) Size matters! How thumbnail number, size, and motion influence mobile video retrieval. In: Lee KT, Tsai WH, Liao HY, Chen T, Hsieh JW, Tseng CC (eds) Advances in multimedia modeling, lecture notes in computer science. doi:10.1007/978-3-642-17829-0_22, vol 6524. Springer Berlin Heidelberg, pp 230–240

[26] Hürst W, Snoek CG, Spoel WJ, Tomin M (2010) Keep moving!: Revisiting thumbnails for mobile video retrieval. In: Proceedings of the international conference on multimedia, MM ’10. doi:10.1145/1873951.1874124. ACM, New York, pp 963–966

[27] Hürst W, van de Werken R, Hoet M (2015) A storyboard-based interface for mobile video browsing. In: He X, Luo S, Tao D, Xu C, Yang J, Hasan M (eds) MultiMedia modeling, lecture notes in computer science. doi:10.1007/978-3-319-14442-9_25, vol 8936. Springer International Publishing, pp 261–265

[28] Jegou H, Douze M, Schmid C, P, P (2010) Aggregating local descriptors into a compact image representation. In: IEEE conference on computer vision and pattern recognition (CVPR), 2010. doi:10.1109/CVPR.2010.5540039. IEEE, pp 3304–3311

[29] Johnson S (1967). Hierarchical clustering schemes. Psychometrika.

[30] Kruliš M, Lokoč J, Skopal T (2013) Efficient extraction of feature signatures using multi-gpu architecture. In: MMM (2), pp 446–456

[31] Le DD, Lam V, Ngo TD, Tran VQ, Nguyen VH, Duong DA, Satoh S (2013) Nii-uit-vbs: a video browsing tool for known item search. In: Li S, Saddik AE, Wang M, Mei T, Sebe N, Yan S, Hong R, Gurrin C (eds) MultiMedia modeling, lecture notes in computer science, vol 7733. Springer International Publishing, pp 547–549

[32] Lin YC, Hu MC, Cheng WH, Hsieh YH, Chen HM (2012) Actions speak louder than words: Searching human action video based on body movement. In: Proceedings of the international conference on multimedia, MM ’12. doi:10.1145/2393347.2396432. ACM, New York, pp 1261–1262

[33] Lokoč J, Blažek A, Skopal T (2014) Signature-based video browser. In: Gurrin C, Hopfgartner F, Hürst W, Johansen H, Lee H, O’Connor N (eds) MultiMedia modeling, lecture notes in computer science, vol 8326. Springer International Publishing, pp 415–418

[34] Mei T, Rui Y, Li S, Tian Q (2014). Multimedia search reranking: a literature survey. ACM Comput Surv.

[35] Moumtzidou A, Avgerinakis K, Apostolidis E, Markatopoulou F, Apostolidis K, Mironidis T, Vrochidis S, Mezaris V, Kompatsiaris Y, Patras I (2015) Verge: a multimodal interactive video search engine. In: He X, Luo S, Tao D, Xu C, Yang J, Hasan M (eds) MultiMedia modeling, lecture notes in computer science. doi:10.1007/978-3-319-14442-9_25, vol 8936. Springer International Publishing

[36] Ngo TD, Nguyen VH, Lam V, Phan S, Le DD, Duong DA, Satoh S (2014) Nii-uit: a tool for known item search by sequential pattern. In: Gurrin C, Hopfgartner F, Hurst W, Johansen H, Lee H, O’Connor N (eds) MultiMedia modeling, lecture notes in computer science, vol 8326. Springer International Publishing, pp 419–422

[37] Ngo TD, Nguyen VT, Nguyen VH, Le DD, Duong Duc A, Satoh S (2015) Nii-uit browser:a multimodal video search system. In: He X, Luo S, Tao D, Xu C, Yang J, Hasan M (eds) MultiMedia modeling, lecture notes in computer science, vol 8936. Springer International Publishing, pp 278–281

[38] Rossetto L, Giangreco I, Schuldt H (2014) Cineast: A multi-feature sketch-based video retrieval engine. In: IEEE international symposium on multimedia (ISM), 2014. IEEE, pp 18–23

[39] Rossetto L, Giangreco I, Schuldt H, Dupont S, Seddati O, Sezgin M, Sahillioglu Y (2015) Imotion - a content-based video retrieval engine. In: He X, Luo S, Tao D, Xu C, Yang J, Hasan M (eds) MultiMedia modeling, lecture notes in computer science, vol 8936. Springer International Publishing , pp 261–265

[40] Rubner Y, Tomasi C (2001). Perceptual metrics for image database navigation.

[41] Schoeffmann K (2014). A user-centric media retrieval competition: The video browser showdown 2012-2014. IEEE MultiMedia.

[42] Schoeffmann K, Ahlström D, Bailer W, Cobârzan C, Hopfgartner F, McGuinness K, Gurrin C, Frisson C, Le DD, Del Fabro M, Bai H, Weiss W (2014). The video browser showdown: a live evaluation of interactive video search tools. IJMIR.

[43] Schoeffmann K, Boeszoermenyi L (2009) Video browsing using interactive navigation summaries. In: 7th international workshop on content-based multimedia indexing, 2009. CBMI ’09, pp 243–248

[44] Schoeffmann K, Cobȧrzan C (2013) An evaluation of interactive search with modern video players. In: IEEE international conference on multimedia and expo workshops (ICMEW), 2013. IEEE, pp 1–4

[45] Schoeffmann K, Hopfgartner F (2015) Interactive video search. In: Proceedings of the 23rd annual ACM conference on multimedia conference (MM ’15). doi:10.1145/2733373.2807417. ACM, New York, pp 1321–1322

[46] Schoeffmann K, Hopfgartner F, Marques O, Boeszoermenyi L, Jose JM (2010) Video browsing interfaces and applications: a review. SPIE Rev 1(1):018004. doi:10.1117/6.0000005. http://link.aip.org/link/?SV2/1/018004/1

[47] Schoeffmann K, Hudelist MA, Huber J (2015) Video interaction tools: a survey of recent work. ACM Comput Surv 1–36. Accepted for publication

[48] Schoeffmann K, Taschwer M, Boeszoermenyi L (2010) The video explorer: a tool for navigation and searching within a single video based on fast content analysis. In: MMSys’10. doi:10.1145/1730836.1730867. ACM, pp 247–258

[49] Sun Q, Hürst W (2008). Video browsing on handheld devices - interface designs for the next generation of mobile video players. IEEE Multimedia.

[50] Worring M, Sajda P, Santini S, Shamma DA, Smeaton AF, Yang Q (2012). Where is the user in multimedia retrieval?. IEEE MultiMedia.

[51] Zhang Z, Albatal R, Gurrin C, Smeaton Alan F (2015) Interactive known-item search using semantic textual and colour modalities. In: He X, Luo S, Tao D, Xu C, Yang J, Hasan M (eds) MultiMedia modeling, lecture notes in computer science, vol 8936. Springer International Publishing, pp 282–286

